# Circulating insulin-like growth factor I modulates mood and is a biomarker of vulnerability to stress: from mouse to man

**DOI:** 10.1038/s41398-018-0196-5

**Published:** 2018-08-01

**Authors:** A. Santi, M. Bot, A. Aleman, B. W. J. H. Penninx, I. Torres Aleman

**Affiliations:** 1 Cajal Institute, Madrid, Spain; 2 Ciberned, Madrid, Spain; 3 Department of Psychiatry, VU University Medical Center and GGZ inGeest, Amsterdam Public Health Research Institute, Amsterdam Neuroscience, Amsterdam, The Netherlands; 4 Department of Neuroscience, University of Groningen and University Medical Center Groningen, Groningen, The Netherlands

## Abstract

Individual susceptibility to anxiety disorders after maladaptive responses to stress is not well understood. We now report that while exploring stress responses in mice after traumatic brain injury (TBI), a condition associated to stress susceptibility, we observed that the anxiogenic effects of either TBI or exposure to life-threatening experiences (predator) were blocked when both stressors were combined. Because TBI increases the entrance into the brain of serum insulin-like growth factor I (IGF-I), a known modulator of anxiety with a wide range of concentrations in the human population, we then determined whether circulating IGF-I is related to anxiety measures. In mice, anxiety-like responses to predator were inversely related to circulating IGF-I levels. Other indicators of mood regulation such as sensitivity to dexamethasone suppression and expression levels of blood and brain FK506 binding protein 5 (FKBP5), a co-chaperone of the glucocorticoid receptor that regulates its activity, were also associated to circulating IGF-I. Indeed, brain FKBP5 expression in mice was stimulated by IGF-I. In addition, we observed in a large human cohort (*n* = 2686) a significant relationship between plasma IGF-I and exposure to recent stressful life events, while FKBP5 expression in blood cells was significantly associated to plasma IGF-I levels. Collectively, these data indicate that circulating IGF-I appears to be involved in mood homeostasis across different species. Furthermore, the data in mice allow us to indicate that IGF-I may be acting at least in part by modulating FKBP5 expression.

## Introduction

Affective disorders affect many people worldwide and currently represent an increasing health burden^1^. Dysregulation of stress homeostasis is considered a key step in development of these disorders, and is a characteristic of conditions such as post-traumatic stress disorder (PTSD) and associated anxiety disorders^2^. PTSD is a heritable condition, and although genetic risk factors are slowly being unveiled^3^, vulnerability to PTSD, and to maladaptive stress responses in general, is not yet fully understood. Indeed, responses to stress show a wide individual variation^4^. For instance, only a subset of combatants develops PTSD after exposure to similar war conditions^5^. One factor proposed to contribute to development of PTSD in war fighters/car accident survivors is traumatic brain injury (TBI)^5–7^. However, because there are also reports that TBI may protect against PTSD, it has been hypothesized that when damage encompasses brain areas putatively involved in stress responses such as prefrontal cortex and amygdala, PTSD does not develop^6,8^. The latter observation opens the possibility to explore endogenous mechanisms of protection against PTSD related to TBI, and probably of vulnerability to stress in general.

Among many potential candidates of endogenous protective mechanisms we focused on two: insulin-like growth factor I (IGF-I) and FK506 binding protein 5 (FKBP5). The former is an abundant circulating hormone mostly produced by the liver^9^. Blood IGF-I levels in the normal population show a wide range of values, with individuals in the lower or higher ends showing greater susceptibility to different diseases^10–15^. Some, but not all studies, show that IGF-I is increased in affective disorders^16,17^. In addition, IGF-I has been documented as a mood regulator^18,19^, as for example through its potent anxiolytic actions^20^. Furthermore, we recently observed that TBI induces the entrance of serum IGF-I into the brain^21^. Thus, IGF-I may be involved in mood regulation after TBI.

In turn, FKBP5 is an Hsp90-associated co-chaperone that regulates the responsiveness of steroid hormone receptors, including glucocorticoids, major regulators of mood and PTSD responses^22^. FKBP5 has been proposed to mediate environmental and genetic interactions in mood regulation^23^, and is associated to stress resilience, PTSD risk, and affective disorders^24–26^. Thus, FKBP5 may also be involved in modulation of stress responses after TBI.

Based on the above observations we consider likely that circulating IGF-I may modulate mood and speculate that FKBP5 may be involved in the actions of IGF-I. Thus, in the present work, we analyzed anxiety-like responses to TBI and/or predator exposure, and FKBP5 expression in mice with varying levels of serum IGF-I. We also performed a cross-sectional study in humans categorized according to serum IGF-I levels to analyze a possible relationship between serum IGF-I and measures of stress exposure and FKBP5 expression. We found that serum IGF-I levels impact on anxiety responses and modulates brain expression of FKBP5 in mice, whereas in humans, lower levels of circulating IGF-I significantly correlated with higher occurrence of stressful events and lower FKBP5 expression.

## Results

### Exploring a link between life-threatening events and brain trauma in anxiety-like behavior in mice

**Fig. 1:**
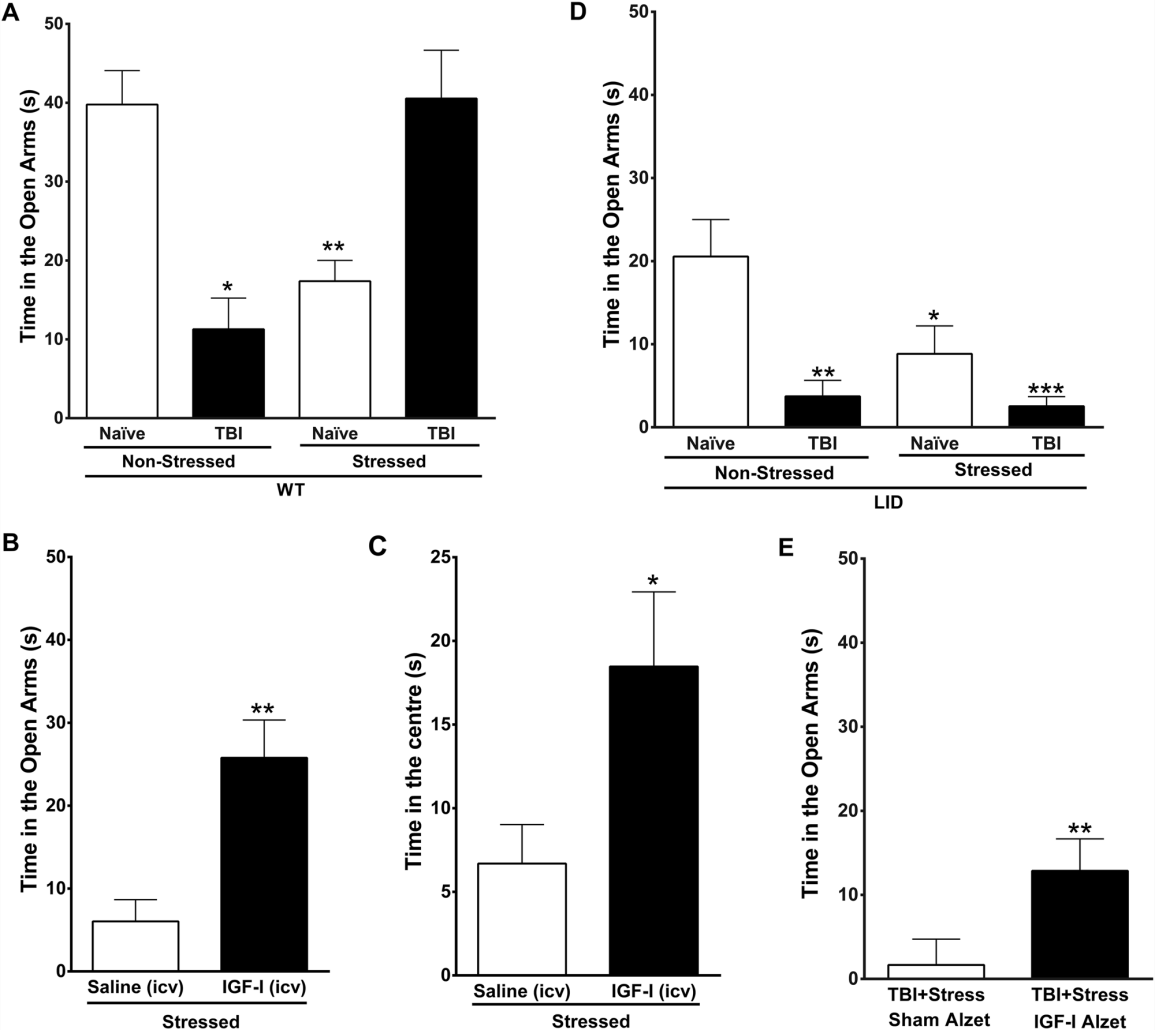
A role for circulating IGF-I in anxiety. **a** Submitting mice to either TBI or exposure to a predator (stressed mice) significantly increased anxiety levels as measured 1 week after in the elevated plus maze (EPM) as time spent in the open arms. However, when both stressors are combined, anxiety is not increased over basal levels (interaction between stress and injury F(_1,111_) = 20.174, *p* < 0.0001; *n* = 38, 10, 32, 35 respectively). **b** Intracerebroventricular administration of IGF-I to intact mice attenuates anxiety responses to predator exposure as determined in the EPM (*t* = 3.606, df = 13, *n* = 7 and 8). **c** Similarly reduced anxiety was seen when placing the mice in an open field, as IGF-I-treated mice showed greater time in the center of the arena (*t* = 2.242, df = 13, *n* = 7 and 8). **d** Combined action of TBI and a life-threatening event (predator exposure) on anxiety levels in mice with low serum IGF-I (LID mice). These mice showed greater basal anxiety as compared to control mice (see panel **a**), enhanced anxiety after TBI injury, and even greater anxiety when TBI was combined to predator exposure (no significant interaction; effects of injury F(_1,46_) = 15.15; *p* = 0.0003 and stress F(_1,46_) = 4.71; *p* = 0.0352; *n* = 12, 11, 13, 14, respectively). **e** Treatment of LID mice with systemic IGF-I ameliorated anxiety after combined TBI and rat exposure (Mann−Whitney *p* = 0.0012; *n* = 7 in both groups). *p < 0.05; **p < 0.01, and ***p < 0.001 vs. respective controls in this and following figures

Exposure of naïve mice to life-threatening events such as a rat, a natural predator that elicits a marked increase in serum levels of the stress hormone corticosterone (Suppl Figure A), increased their anxiety levels, as shown by a decrease in the time spent in the open arms of the elevated plus maze (Fig. 1a). Similarly, physical stress produced by a TBI also enhanced anxiety in wild-type mice (Fig. 1a). However, when both stressors (predator exposure + TBI) are combined, as is often the case for PTSD-triggering scenarios^6,7^, the time spent in the open arms of the elevated plus maze (as a measure of anxiety-like behavior) was not different from nonstressed, nonlesioned intact mice (Fig. 1a). Differences in time spent in open arms were not related to differences in exploratory activity of the experimental groups as all of them showed similar number of entries in the arms (Supp Figure B).

Next, we determined whether TBI-induced entrance of serum IGF-I into the brain^21^ could ameliorate stress responses after combined TBI and predator exposure, as IGF-I modulates mood^20^. First, we administered intracerebroventricular IGF-I to intact mice to mimic the increase in IGF-I produced by TBI^21^, and 2 days later we submitted them to predator stress. We observed that treatment with IGF-I, but not its vehicle, significantly attenuated the stress response to predator exposure, as determined both by reduced anxiety in the elevated plus maze (Fig. 1b) and in the open field (Fig. 1c). To confirm a role of IGF-I in anxiolysis, we then determined anxiety levels after combined exposure to TBI and predator in mutant mice with low serum IGF-I levels (LID mice), that show only a modest increase in brain IGF-I levels after TBI^21^. We observed that LID mice displayed an enhanced increase in anxiety after exposure to predator and TBI (Fig. 1d). This is in marked contrast with wild-type mice exposed both to TBI + predator, that did not show changes in anxiety (Fig. 1a, right-most bar). As normalization of brain function in LID mice is possible by treating them with systemic IGF-I^27^, we analyzed if their exaggerated anxiety response could be attenuated after systemic IGF-I treatment. Indeed, IGF-I administration to LID mice exerted an anxiolytic effect, as reflected by a significantly greater time spent in the open arms of the EPM (Fig. 1e).

### Serum IGF-I and the HPA axis

**Fig. 2:**
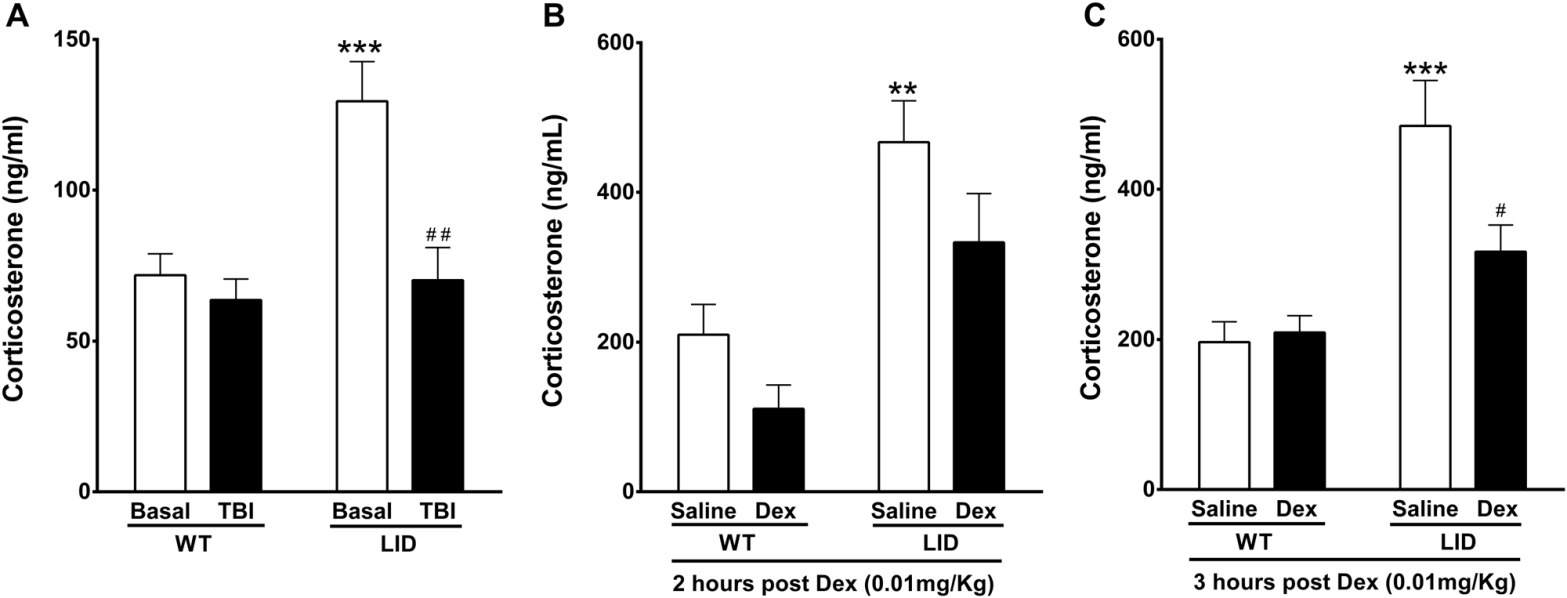
Low serum IGF-I alters corticosterone responses to stress. **a** LID animals show significantly greater basal levels of corticosterone in blood (*p* < 0.001, white bars), as well as a prolonged suppression of this hormone 2 days after exposure to TBI (solid bars, significant interaction between injury and genotype F(_1, 15_) = 41.17; *p* < 0.001; *n* = 8 and 9, in WT and LID respectively). **b**, **c** Suppression of corticosterone by dexamethasone is delayed in LID mice, being stronger at 3 h after dexamethasone treatment (**c**) than 2 h after injection (**b**). Significant effect of treatment (F(_1, 28_) = 5.466; *p* = 0.0268) and genotype (F(_1, 28_) = 23.10; *p* < 0.0001) 2 h after DEX administration (**b**), and significant interaction between genotype and treatment (F(_1, 28_) = 5.300; *p* = 0.0290) 3 h after the injection (*n* = 8 in each group)

The above data pointed to a link between serum IGF-I and anxiety. Accordingly, we observed that LID mice that showed increased anxiety in the elevated plus maze (Fig. 1) had an altered HPA axis. Mice with low serum IGF-I had higher basal serum corticosterone levels (Fig. 2a, bars of basal values), together with higher sensitivity of the HPA axis, as indicated by protracted reduction of serum corticosterone after dexamethasone suppression (Fig. 2b, c). Based on the latter, we examined whether LID mice had also abnormally delayed corticosterone reduction after exposure to the physical stress produced by TBI, that increases corticosterone^28^, and found that corticosterone levels remained low 2 days after injury (Fig. 2a).

**Fig. 3:**
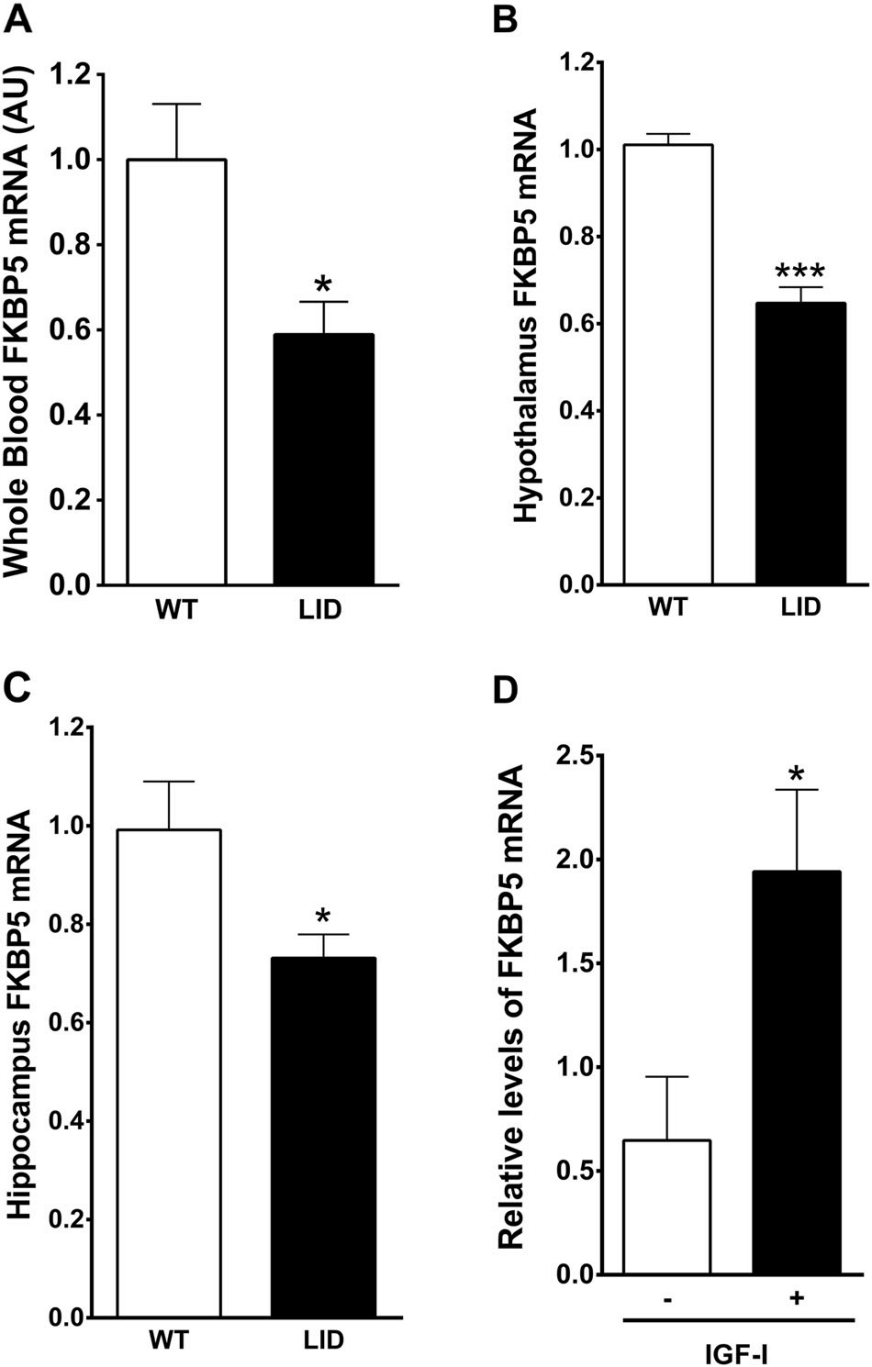
FKBP5 is regulated by IGF-I. **a** LID mice with low serum IGF-I have lower levels of FKBP5 mRNA in blood (*t* = 2.82; df = 9; *p* = 0.02; *n* = 5 and 6, respectively); **b** in hypothalamus (*t* = 8.503; df = 21; *p* < 0.0001; *n* = 14 and 9, respectively); **c** and in hippocampus (*t* = 2.7; df = 12; *p* = 0.0193; *n* = 5/9, respectively). **d** Treatment of mixed hypothalamic cultures with IGF-I increases expression of FKBP5 (*t* = 2.587; df = 8; *p* = 0.0323; *n* = 5 for both groups)

**Fig. 4:**
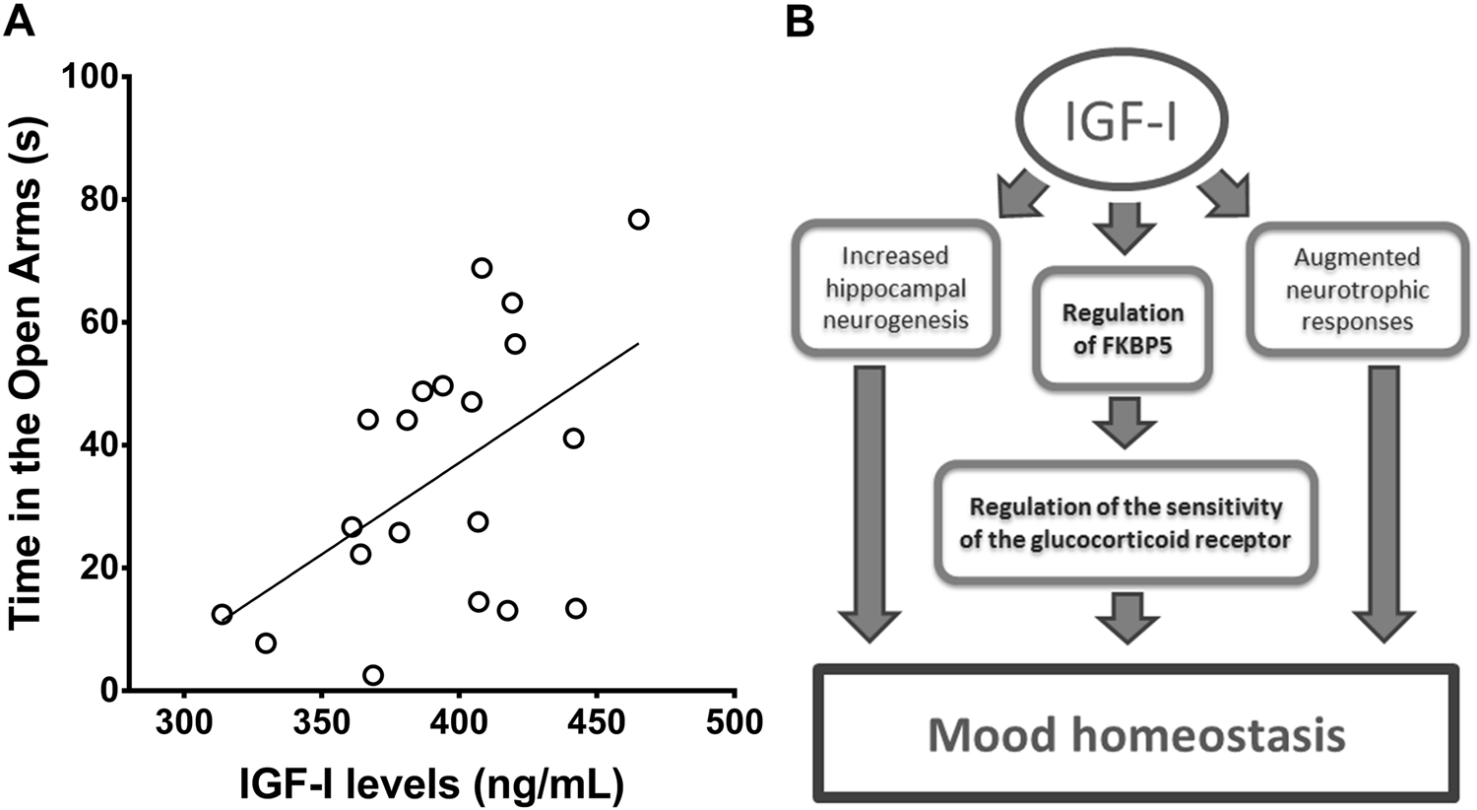
IGF-I and anxiety. **a** A significant correlation was seen between serum IGF-I levels and anxiety measured in the EPM (F (_1,18_) = 6.483; *p* = 0.0203; *n* = 20). **b** Proposed mechanisms mediating serum IGF-I effects on mood. Circulating IGF-I potentiates hippocampal neurogenesis and neurotrophic signaling, as already published^27,39^, while it also stimulates FKBP5 in brain, modulating in this way the activity of the brain glucocorticoid receptor that eventually impacts on mood homeostasis

To start exploring mechanisms connecting IGF-I with the regulation of the HPA axis we determined the levels of the co-chaperone FKBP5, an important determinant of the activity of the glucocorticoid receptor that is related to PTSD risk^24^ and stress-sensitive brain systems^29^. We found that low serum IGF-I in LID mice parallels lower levels of expression of FKBP5 in blood, hypothalamus, and hippocampus (Fig. 3a−c). Furthermore, IGF-I stimulated expression of FKBP5 in mixed hypothalamic cultures (Fig. 3d). Since serum IGF-I likely impacts on the activity of the HPA axis by modulating FKBP5, it may help assess the activity of this neuroendocrine axis. Indeed, in control mice, serum IGF-I levels correlated with anxiety levels, as measured in the elevated plus maze (Fig. 4a).

Based on these observations in mice, and because of its potential clinical utility as a biomarker of vulnerability to stress, we determined in a large human cohort (Netherlands Study of Depression and Anxiety, NESDA) whether plasma IGF-I levels were related with measures of stress exposure, HPA activity, and FKBP5. Data were available for up to 2686 NESDA participants (mean age 41.7 (SD 13.0) years, and 66% females). Table 1 shows the associations of IGF-I with number of life events in the past year, childhood trauma, dexamethasone suppression ratio, and FKBP5 expression. We found a positive association between the lowest quintile of IGF-I (compared to the middle quintile) and number of life events, suggesting that persons that were in the lowest quintile of IGF-I had experienced 1.17 times more life events than persons in the middle quintile. IGF-I was not related to any of the other stress measures. Furthermore, IGF-I as a continuous measure was positively associated with averaged FKBP5 expression.

**Table 1:** Association of IGF-I with clinical and gene expression variables in a large-scale human sample

		Outcomes			
		Number of life events	Childhood trauma (y/n)	Dexamethasone suppression ratio^a^	Mean FKBP5 expression
		*n* = 2686	*n* = 2676	*n* = 1706	*n* = 1811
		Poisson regression	Logistic regression	Linear regression	Linear regression
Model		RR (95% CI), *p*	OR (95% CI), *p*	b (95% CI), *p*	b (95% CI), *p*
	Independent variable				
1.	IGF-I (continuous variable)	0.997 (0.991−1.002), 0.24	1.00 (0.99−1.02), 0.58	0.000 (−0.001 to 0.002), 0.56	**0.003 (0.000−0.005)**, **0.03**
2.	IGF-I quintiles				
	Q1	**1.17 (1.03–1.32)**, **0.02**	0.94 (0.73–1.20), 0.59	−0.019 (−0.053 to 0.015), 0.27	−0.033 (−0.085 to 0.018), 0.20
	Q2	0.99 (0.87–1.12), 0.83	0.88 (0.69–1.13), 0.31	−0.006 (−0.039 to 0.028), 0.75	0.044 (−0.006 to 0.094), 0.08
	Q3 (reference)				
	Q4	0.98 (0.86–1.12), 0.79	0.80 (0.62–1.02), 0.07	0.002 (−0.032 to 0.035), 0.92	−0.002 (−0.054 to 0.049), 0.93
	Q5	1.00 (0.88–1.14), 0.99	1.11 (0.87–1.42), 0.40	−0.006 (−0.039 to 0.028), 0.74	0.036 (−0.015 0.087), 0.17

Bold values indicate significant differences

All analysis are adjusted for: sex, age, age^2^, education level, smoking, BMI, physical activity, glucose

*CI* confidence interval, *IGF-I* insulin-like growth factor I, *OR* odds ratio, *RR* rate ratio

## Discussion

The present observations support the hypothesis that circulating IGF-I modulates mood at least in part by modulating FKBP5 activity. While prior observations suggested that IGF-I might be involved in affective disorders (see introduction), these results reveal a specific role for circulating IGF-I in mood homeostasis, as gathered from experimental data in mice and epidemiological data from a large human cohort. Since circulating IGF-I is mostly produced by the liver^9^, this suggests that this peripheral organ is involved in mood regulation. Indeed, glucocorticoids, major modulators of mood^30^, are well-known regulators of serum IGF-I levels^31–33^, which in turn also modulate glucocorticoid production^34^.

We have seen that in mice, serum IGF-I is an important determinant of anxiety-like responses. Assessment of underlying mechanisms indicated that circulating IGF-I impacts on the activity of the HPA axis, probably in part by regulating FKBP5 expression, which is also regulated by glucocorticoids^35^, and in this way regulates anxiety. Analysis of this relationship in the NESDA human cohort showed that persons with greater vulnerability to stress, as determined by reported stressful occurrences but not by other measures of stress vulnerability, had the lowest levels of serum IGF-I. In turn, IGF-I also correlated with FKBP5 levels in this human cohort. Previously, the NESDA study showed positive association between IGF-I and depressive and anxiety disorders when controlling for antidepressant medication use^17^.

Although limitations of NESDA need to be accounted for, including that persons with a depressive and/or anxiety disorder were overrepresented, no measures of PTSD were done in the baseline study and other, maybe more relevant stress vulnerability measures were not included, we consider that these data, in conjunction with the results in mice allow us to suggest that measurement of serum IGF-I may help in the clinical assessment of susceptibility to stress. While more studies are needed, these results could help explain individual vulnerability to stress situations after TBI, with patients with lower IGF-I levels being at greater risk. This is an important point, since the relationship between TBI and PTSD risk is not entirely clear^6^. Because IGF-I directly modulates FKBP5 in brain cells, and serum IGF-I can enter into the brain in a regulated fashion^36^, the reported anxiolytic actions of pharmacological boosting of IGF-I^37^ can be explained by the present observations. Indeed, we provide direct evidence of anxiolysis by IGF-I in mice. Ultimately, these observations could help to clarify the relationship between TBI and PTSD, as subjects with lower serum IGF-I/FKBP5 will likely show greater susceptibility to PTSD.

In this regard, our work in experimental models allow us to hypothesize that TBI-induced uptake of serum IGF-I by the brain will be reduced in individuals with low serum IGF-I, as seen previously in mice^21^. Reduced entrance of IGF-I will impact on brain glucocorticoid receptor sensitivity, that is altered after TBI^38^, at least in part through regulation of brain FKBP5 expression, as evidenced by exacerbated stress responses in mice with low serum IGF-I (LID mice). A role for IGF-I is corroborated by our observation that intracerebroventricular administration of this growth factor in control mice greatly attenuates subsequent stress responses. Other potential anxiolytic actions of IGF-I, including promotion of hippocampal neurogenesis and growth factor responses^39,40^, will be also reduced if insufficient IGF-I is available. In mice with low serum IGF-I, the observed prolonged corticosterone reduction after TBI will likely interfere with the subsequent stress response to predator exposure, leading to exacerbated anxiety. In humans, as no correlation of circulating IGF-I with dexamethasone suppression was observed, other mechanisms downstream of low IGF-I may be involved.

The association between serum IGF-I and measures of mood regulation (anxiety, etc) might be bi-directional. That is, serum IGF-I and mood may influence each other. In this regard, it is well-known that glucocorticoids, that are altered in mood disorders, and IGF-I influence each other^31,34^,. Based on the observations in LID mice, reduced serum IGF-I (as a result of genetic ablation of the liver IGF-I gene) is associated to enhanced anxiety and diminished capacity to cope with stress. In turn, early stressful events may lead to lower serum IGF-I levels in humans, as was recently reported in infant rats^41^. Indeed, lower socioeconomic status correlates with lower serum IGF-I^42^, probably in part due to poorer nutrition^43^, but probably also because of increased exposure to stress conditions associated to low income^44^. Intriguingly, lower serum IGF-I levels have been shown to be associated to lower social rank/sociability across the phylogenetic tree^45–48^. Furthermore, prolonged lower serum IGF-I leads to depressive states in mice^19^. Conversely, it has been observed that enhanced resilience to stress in adult mice reared under enriched environment is mediated by serum IGF-I^18^. However, serum IGF-I levels are higher in affective disorders in humans^16,17^, which may indicate an adaptive/resistant process, but this remains to be explored. At any rate, our observations fit well with a previously noted role of serum IGF-I in mood homeostasis.

In summary, we now provide evidence that the association of TBI with subsequent anxiety-like behaviors may depend on serum IGF-I levels prior to insult. Serum IGF-I levels are likely to influence the impact of TBI on mood homeostasis because brain IGF-I levels after TBI increase in proportion to circulating IGF-I levels^21^. Of broader consequence, IGF-I levels may contribute, together with other relevant variables, to a “risk profile” of vulnerability to stress in populations at risk, or even in the general population, with low levels positing as a risk factor to stress vulnerability. At any rate, larger studies in humans are needed to establish whether blood IGF-I levels may serve as a biomarker of stress vulnerability. Of note, as serum IGF-I levels can be increased by pharmacological, behavioral, and nutritional intervention^37,43,49^, vulnerability to stress may be ultimately modulated for better through multifactorial approaches.

## Materials and methods

### Animals

Adult male control (C56BL/6JolaHsd; 28–35 g) and mutant mice with low serum IGF-I (LID mice; 27–38 g), and adult male and female Wistar rats (200–450 g) obtained from the Cajal Institute were used. Mice were housed in standard cages (48 × 26 cm^2^; 5 per cage), while rats were housed in standard cages with 1–2 rats per cage. All animals were kept on a light−dark cycle (12–12 h) in a room with constant temperature (22 °C) and humidity, and with food (pellet rodent diet) and water ad libitum. All experimental protocols were performed during the light cycle. Mice were handled for 3 days prior to any experimental manipulations. Animal procedures followed European guidelines (86/609/EEC, European Council Directives) and were approved by the local Bioethics Committee.

### Controlled cortical injury (CCI)

Time-line and experimental design are shown in Suppl Figure G. An electromagnetic stereotaxic impactor (Impact One^TM^, MyNeuroLab, Leica, Germany) was used to induce a mild CCI injury, as previously described in detail elsewhere^21^. Sham injured animals were submitted to the same procedure except for the impact.

### Behavioral tests

#### Predator exposure

C57BL/6 and LID animals were exposed to a rat for 10 min. Mice were assigned to the control or stressed group in a randomized way. A box containing the rat was divided by a plastic mesh leaving the rat in one of the two compartments. Mice were introduced in the other compartment protected by the mesh. This allowed them to see, smell and touch the rat without being harmed. The floor bedding had urine and feces from the rat. Every experimental day each rat was used to stress a maximum of three mice. Control animals were exposed to a similar box with clean bedding and in the opposite compartment, instead of a rat, there was a toy rat.

#### Open field

Mice were introduced in a 42 cm × 42 cm × 30 cm arena (Versamax; AccuScan Instruments, Inc.) for 5 min. Time exploring the center of the open field and total distance was quantified automatically with the software provided by the manufacturer.

#### Elevated plus maze

To assess anxiety-like behavior, animals were introduced in the center of an elevated plus maze for 5 min. The maze was at 40 cm from the floor with two opposing protected (closed) arms of 30 cm (length) × 5 cm (wide) × 15.25 (height), and two opposing unprotected (open) arms (30 × 5 × 0).Time in the open and closed arms, as well as the number of entries in each arm were recorded with an automated video-tracking system (Video Tracking Plus Maze Mouse; Med Associates, USA).

### Dexamethasone suppression test

To test the sensitivity of the HPA axis, mice were injected intraperitoneally with 0.01 mg/kg of dexamethasone (Sigma) or saline. One and a half hours later, animals were introduced in an open field for 10 min to induce a mild corticosterone increase. 2 and 3 h after the injection, blood samples were collected. Results are shown as corticosterone levels of the dexamethasone-treated animals over the control levels of saline-injected mice.

### Blood and tissue collection

As previously described^21^, blood was collected from the submandibular vein to reduce pain and distress. Serum was collected and stored at −20 °C. For FKBP5 mRNA extraction from whole blood, samples were extracted either with a cardiac puncture or from the submandibular vein and immediately homogenized in Trizol LS and frozen until RNA extraction. To obtain the hippocampi and hypothalami, animals were anesthetized with pentobarbital (50 mg/kg, i.p.) and perfused transcardially with saline. Brains were removed, and the areas were dissected and frozen in dry ice. Samples were stored at −80 °C until further use.

### Cell cultures and in vitro assays

Brain samples were extracted from E16-17 mice as previously described^50^. Meninges and blood vessels were removed. Then, the structures were minced with scissors and dissociated with a Pasteur pipette in DMEM-F12. After brief centrifugation at 1000 rpm, the pellet was resuspended in Neurobasal medium supplemented with B27 (Gibco; 395) and Glutamine (Sigma; G3126). Subsequently, neuronal and glia cell cultures were seeded onto six-well plates coated with poly-l-lysine and previously incubated with 10% of FBS in PBS. Plated cells were kept in B27 and glutamine-supplemented Neurobasal, in an atmosphere of 95% O_2_ and 5% CO_2_ at 37 °C. One week after the culture, cells were washed with PBS and fasted for 3 h with B27-free Neurobasal.

### IGF-I administration

#### In vivo

Alzet osmotic mini-pums (Model 1004; USA) were used for chronic administration of hIGF-I (Pre-Protech, USA; 50 µg/kg/day) or the vehicle (saline). Pumps were implanted subcutaneously between the scapulae following the manufacturer’s instructions. The treatment lasted 25 days. Intracerebroventricular infusion of hIGF-I (1 µg/day) was carried out with Alzet osmotic mini-pumps (Model 1002) implanted subcutaneously and attached to a catheter tube and an infusion cannula (Brain infusion kit 3, Alzet) that was positioned with the stereotaxic frame into the left lateral ventricle (−0.6 mm from bregma, 1.4 mm lateral and at 2 mm of depth) according to the manufacturer’s instructions. The treatment lasted 10 days (see Suppl Figure G for time-line of this experiment).

#### In vitro

Following the starving period, cells were treated with rhIGF-I (1 nM) for 24 h. Cells were then tripsinized and frozen at −80 °C until RNA extraction. All experiments were done in triplicate dishes.

### ELISA

#### IGF-I

IGF-I in serum and tissue was determined using a mouse-specific ELISA (R&D Systems, USA) as described^27^, following the manufacturer’s instructions.

#### Corticosterone

Corticosterone in serum was determined using a species-independent enzyme immunoassay kit (DetectX, Arbor Assays) following the manufacturer’s instructions.

### RNA extraction, reverse transcription and Duplex real-time qPCR

Total RNA isolation was carried out following the manufacturer’s instructions with either Trizol (Life Technologies, USA) for the brain tissue, Trizol LS (Life Technologies, USA) for whole blood samples or with the RNA Cell Culture Kit with the MiniQG-80 apparatus for cell samples. RNA retro-transcription and cDNA amplification were performed as previously described^21^ with a FKBP5 TaqMan probe (FAM reporter, Mm00487406_m1), and 18S as endogenous control (VIC reporter, Life Technologies). A modified 2(−ΔΔCT) method^51^ was used to determine the relative mRNA quantity.

### Human data from the NESDA study

To investigate whether IGF-I was associated with stress vulnerability measures and FKBP5 in a human sample, we used data of the Netherlands Study on Depression and Anxiety (NESDA). NESDA is an ongoing longitudinal cohort study on predictors, course and consequences of depressive and anxiety disorders that has been described in detail elsewhere^52^. In brief, the NESDA sample consists of 2981 participants aged 18–65 years, comprising persons with and without depressive and/or anxiety disorders. Exclusion criteria were (1) a primary clinical diagnosis of a psychiatric disorder not under study in NESDA (psychotic disorder, obsessive compulsive disorder, bipolar disorder, or severe addiction disorder), and (2) not being fluent in Dutch. Between September 2004 and February 2007, all participants completed the 4-h baseline assessment at one of the research centers, which included face-to-face interviews, written questionnaires, and biological measurements. The research protocol was approved by the Ethical Committee of the participating centers. All participants provided written informed consent. We excluded persons for whom no blood was sampled in NESDA (*n* = 113), persons whose IGF-I levels could not be determined by the lab (*n* = 145), individuals on growth hormone medication (ATC code H01AC; *n* = 3), antidepressant medication users with no lifetime depressive/anxiety disorder (*n* = 6), and persons with no glucose measure (*n* = 28), which resulted in 2686 participants to be included in the analysis.

### Measurements in NESDA

#### IGF-I

Fasting EDTA plasma samples were collected in the morning during the baseline measurement, and kept frozen at −80 °C until assaying. As described earlier^17^, IGF-I (nmol/l) was assayed centrally by chemiluminescence immunoassay on the Liaison autoanalyzer (DiaSorin, S.p.A., Italy), with intra-assay and interassay coefficient of variation of 8.3 and 11.1%, respectively. IGF-I was studied as a continuous measure. However, as both low and high IGF-I levels might be associated with poor health outcomes, sex- and age-specific quintiles of IGF-I were also computed.

#### Negative life events and childhood trauma in NESDA

The number of negative life events in the last year were measured with the Brugha’s List of Threatening Experiences^53^ and was expressed as number of negative life events in the last year. Childhood trauma was measured with the Childhood Trauma Interview, which measures the presence of emotional neglect, psychological abuse, physical abuse, and sexual abuse before age 16^54^, and was dichotomized into having a traumatic event in childhood vs. having no traumatic event in childhood.

#### Dexamethasone suppression

In a subsample of NESDA participants, saliva was collected to measure dexamethasone suppression. The salivary cortisol measurements have been described before^55^. Briefly, respondents were asked to collect saliva samples at home on a regular (preferably working) day by Salivettes (Sarstedt AG and Co, Nurmbrecht, Germany) at seven time points: upon awakening (T1), 30 min (T2), 45 min (T3), and 60 min (T4) after awakening and in the evening at 2200 hours (T5) and 2300 hours (T6). Immediately after saliva sampling at T6, the cortisol suppression test was carried out by oral administration of a 0.5-mg dexamethasone pill and assessed by cortisol sampling the next morning directly after awakening (T7). Instructions for the saliva sampling prohibited eating, smoking, drinking, or brushing teeth within 15 min before sampling and dental work 24 h before sampling. All samples were refrigerated and returned by mail. During laboratory analysis, Salivettes were centrifuged at 2000 × *g* for 10 min, aliquoted, and stored at −80 °C. Competitive electrochemiluminescence immunoassay (E170, Roche, Basel, Switzerland) was used to measure cortisol levels^56^. The detection limit was 2.0 nmol/l and intra-assay and interassay variability coefficients in the measuring range were <10%. Dexamethasone suppression ratio was calculated by dividing the cortisol levels at awakening on day 1 (T1) by the post-dexamethasone cortisol value at awakening on day 2 (T7).

#### FKBP5 gene expression

To assess FKBP5 gene expression in NESDA, venous whole blood samples were obtained in the morning after overnight fasting. Heparinized whole blood samples were transferred within 20 min of sampling into PAXgene Blood RNA tubes (Qiagen, Valencia, California, USA) and stored at −20 °C before RNA isolation. Gene expression assays were conducted at the Rutgers University Cell and DNA Repository (RUCDR, http://www.rucdr.org). Samples were hybridized to Affymetrix U219 array plates (GeneTitan), which contains 530,467 probes summarized in 49,293 probe sets. Array hybridization, washing, staining, and scanning were carried out in an Affymetrix GeneTitan System per the manufacturer’s protocol. Gene expression data were required to pass standard Affymetrix QC metrics (Affymetrix expression console) before further analysis. We removed probes for further analysis that did not map uniquely to the hg19 (Genome Reference Consortium Human Build 37) reference genome sequence, as well as probes targeting a messenger RNA (mRNA) molecule resulting from a transcription of a DNA sequence containing a single nucleotide polymorphism (based on the dbSNP137 common database), resulting in 423,201 probes, which could be summarized into 44,241 probe sets targeting 18,238 genes. Normalized probe set expression values were obtained using Robust Multi-array Average (RMA) normalization as implemented in the Affymetrix Power Tools software (APT, version 1.12.0, Affymetrix).

Further details on RNA processing procedures have been described before^57,58^. We calculated the mean FKBP5 expression across all six probe sets targeting FKBP5 after adjusting for RNA per plate, position on plate, month and time of blood withdrawal, level of blood hemoglobin, and time between blood withdrawal and RNA extraction.

#### Covariates in NESDA

Gender, age, education level, and smoking status (never, past, current) were measured. Weight and height were measured by trained staff to calculate BMI (kg/m^2^). Physical activity was assessed with the International Physical Activity Questionnaire (IPAQ), and expressed in 1000 metabolic equivalent (MET) minutes per week^59^. Glucose levels were determined using routine standardized laboratorial methods after an overnight fast.

### Statistical analysis

SPSS Statistics 22 (IBM) and GraphPad Prism 6 software (USA) were used to perform statistical analysis. Depending on the number of independent variables and the experimental groups compared, either Student’s *t* test or Mann−Whitney *U* test (two groups parametric or nonparametric) and one-way or two-way ANOVAs (for more than two groups) followed by Bonferroni’s multiple comparison test as a post-hoc test were performed. Results are shown as mean ± standard error (SEM) and *p* values coded as follows: *p < 0.05, **p < 0.01, ***p < 0.001. Linear regression analyses were carried out for the study of the association between IGF-I and anxiety in mice. Sample size for each experiment was chosen based on previous experience and aimed to detect at least a *p* < 0.05 in the different tests applied. All animals were included in the statistical analysis, without any exclusion criteria. Animals were included in each experimental group randomly by the researcher, without blinding.

In NESDA, different types of regression analyses were performed to study the associations between IGF-I (independent variable), and each of the stress vulnerability measures and FKBP5 (dependent variables). A Poisson regression model (which provides a rate ratio) was used for the number of life events; a logistic regression model was used for childhood trauma (y/n), and linear regression models were used for dexamethasone suppression ratio and FKBP5 expression. Due to the skewed distribution, dexamethasone suppression ratio was log-transformed before being entered into the regression model. All analyses were first done with IGF-I as continuous variable (model 1) and were then repeated with the sex- and age-specific IGF-I quintiles as independent variable (with Q3 as reference category, model 2). All models were adjusted for gender, age, education level, smoking, body mass index, physical activity and glucose. Analyses were done in IBM SPSS version 24, and a *p* < 0.05 was considered statistically significant.

## Electronic supplementary material

Suppl Fig 1
